# Refractory disfiguring lymphocytoma cutis with rapid and dramatic response to upadacitinib

**DOI:** 10.1016/j.jdcr.2026.09.031

**Published:** 2026-09-23

**Authors:** Eunice Y. Ju, Nidhi Kuchimanchi, Sarah P. Pourali, Alejandro Gru, Jennifer A. DeSimone

**Affiliations:** a University of Virginia School of Medicine, Charlottesville, Virginia; b Department of Dermatology, Columbia University Medical Center, New York, New York; c Inova Schar Cancer Institute, Fairfax, Virginia

**Keywords:** BCL6, benign B-cell lymphoproliferative disorder, CD10, cutaneous lymphoid hyperplasia, cutaneous pseudolymphoma, JAK inhibitor, Ki-67, lymphocytoma cutis, refractory, upadacitinib

## Introduction

Lymphocytoma cutis (LC), also known as cutaneous lymphoid hyperplasia or cutaneous pseudolymphoma, is a rare, benign B-cell lymphoproliferative disorder. Most cases of LC are idiopathic, although triggers include *Borrelia burgdorferi* infection, arthropod bites, vaccinations, tattoos, and piercings.^1^ LC is characterized by erythematous to violaceous papules, plaques, or nodules. Although LC is considered a reactive process, its clinical and histopathologic features can overlap with primary cutaneous B-cell lymphoma, making accurate diagnosis essential.^2^

Management of LC depends on triggers and disease distribution.^3^ Localized lesions often respond to treatments such as intralesional corticosteroids, cryosurgery, or surgical excision.^4^ Disseminated disease may follow a more chronic course and present with greater therapeutic challenges, often responding incompletely to hydroxychloroquine or rituximab, with frequent relapses.^1^^,^^4^

Janus kinase (JAK) inhibitors are immunomodulatory drugs that target cytokine signaling pathways involved in inflammatory and lymphoproliferative processes and have demonstrated efficacy across dermatologic conditions.^5^^,^^6^ However, their role in benign B-cell lymphoproliferative disorders has not been well-characterized. Importantly, JAK inhibitors carry boxed warnings for malignancy, including lymphoma, which raises considerations when used in disorders involving B-cell proliferation.^7^ Although upadacitinib has been reported in isolated cases of cutaneous pseudolymphoma, its use in LC has not previously been described. Here, we describe a patient with refractory LC who achieved significant clinical improvement with upadacitinib after failing multiple conventional therapies.

## Case report

A 50-year-old healthy male presented with a 10-year history of scattered, firm, pruritic violaceous papules and nodules on the head and extremities (Fig 1). Lesions were slowly progressive and disfiguring. No lymphadenopathy was noted on examination.

**Fig 1:**
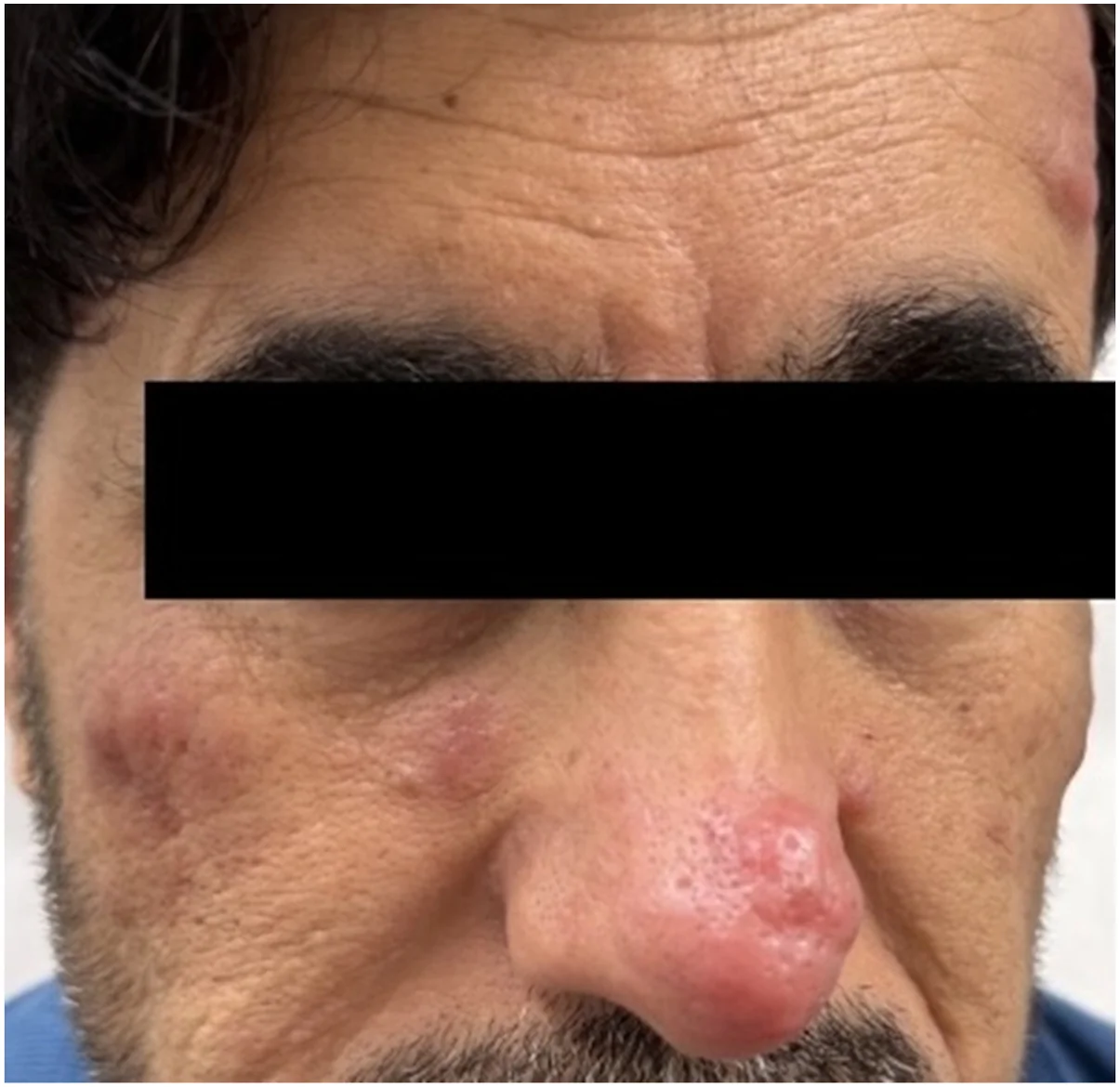
Lymphocytoma cutis. Clinical presentation at initial visit showing coalescent erythematous to violaceous papules and nodules on the forehead, temples, and nose.

Two prior biopsies of the left temple performed in 2018 demonstrated findings consistent with LC. Additional biopsies performed in Afghanistan reportedly showed no evidence of clonal immunoglobulin heavy chain gene rearrangement. Systemic evaluation with computed tomography of the chest, abdomen, and pelvis demonstrated no evidence of systemic disease.

Over the disease course, the patient trialed multiple therapies, including topical, intralesional, and oral pulsed corticosteroids, as well as hydroxychloroquine, doxycycline, and 2 courses of low-dose radiation therapy, all without clinical improvement.

Laboratory studies revealed a normal complete blood count with differential, comprehensive metabolic panel, lipid panel, and leukemia/lymphoma panel. Lyme serologies were negative. A repeat shave biopsy at our clinic showed dense dermal lymphocytic infiltrates with reactive germinal centers (Fig 2). Immunohistochemical staining showed CD20 positive B-cells. RNA in situ hybridization for kappa and lambda light chains showed a polyclonal pattern, supporting a reactive lymphoid process. Reactive germinal centers stained positive for CD10 and BCL6 but negative for BCL2, while Ki-67 demonstrated a high proliferation index within the germinal centers. Tissue flow cytometry was not performed. Overall, the findings favored a reactive lymphoid process rather than cutaneous B-cell lymphoma.

**Fig 2:**
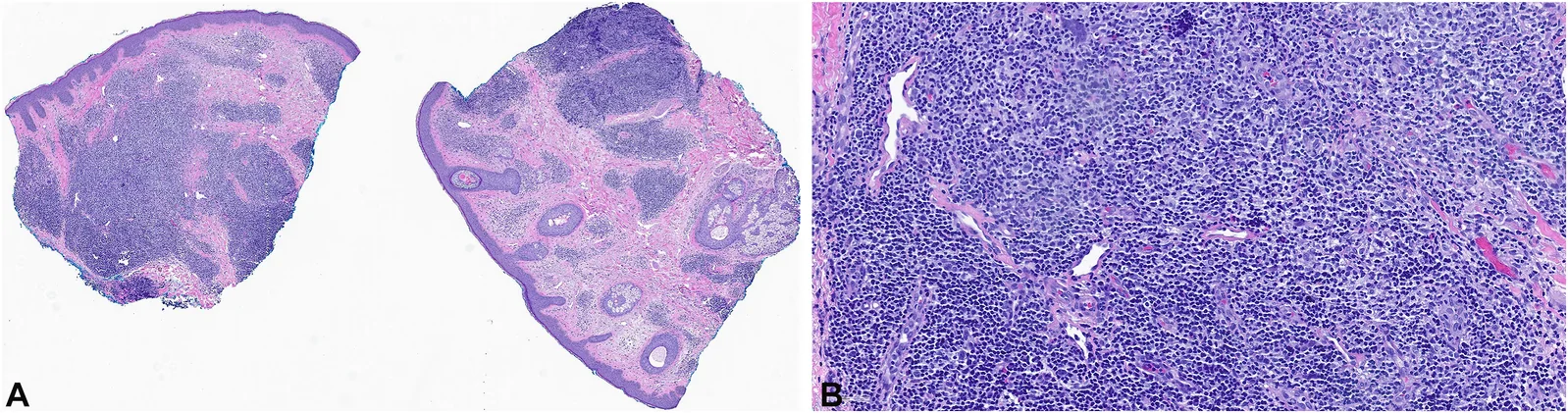
Left pre-auricular biopsy findings. **A,** Dense nodular dermal lymphoid infiltrates with multiple well-formed germinal centers (hematoxylin and eosin stain, 10×). **B,** Reactive germinal center in same tissue fragment as A (hematoxylin and eosin stain, 40×).

Given the patient’s refractory disease and preference to avoid further radiation therapy, treatment with upadacitinib 15 mg daily was initiated after standard pretreatment screening for upadacitinib was performed. At 12-week follow-up, the patient demonstrated near-complete resolution of the lesions, with residual post-inflammatory hyperpigmentation (Fig 3). The patient reports continued improvement and is tolerating the therapeutic regimen well.

**Fig 3:**
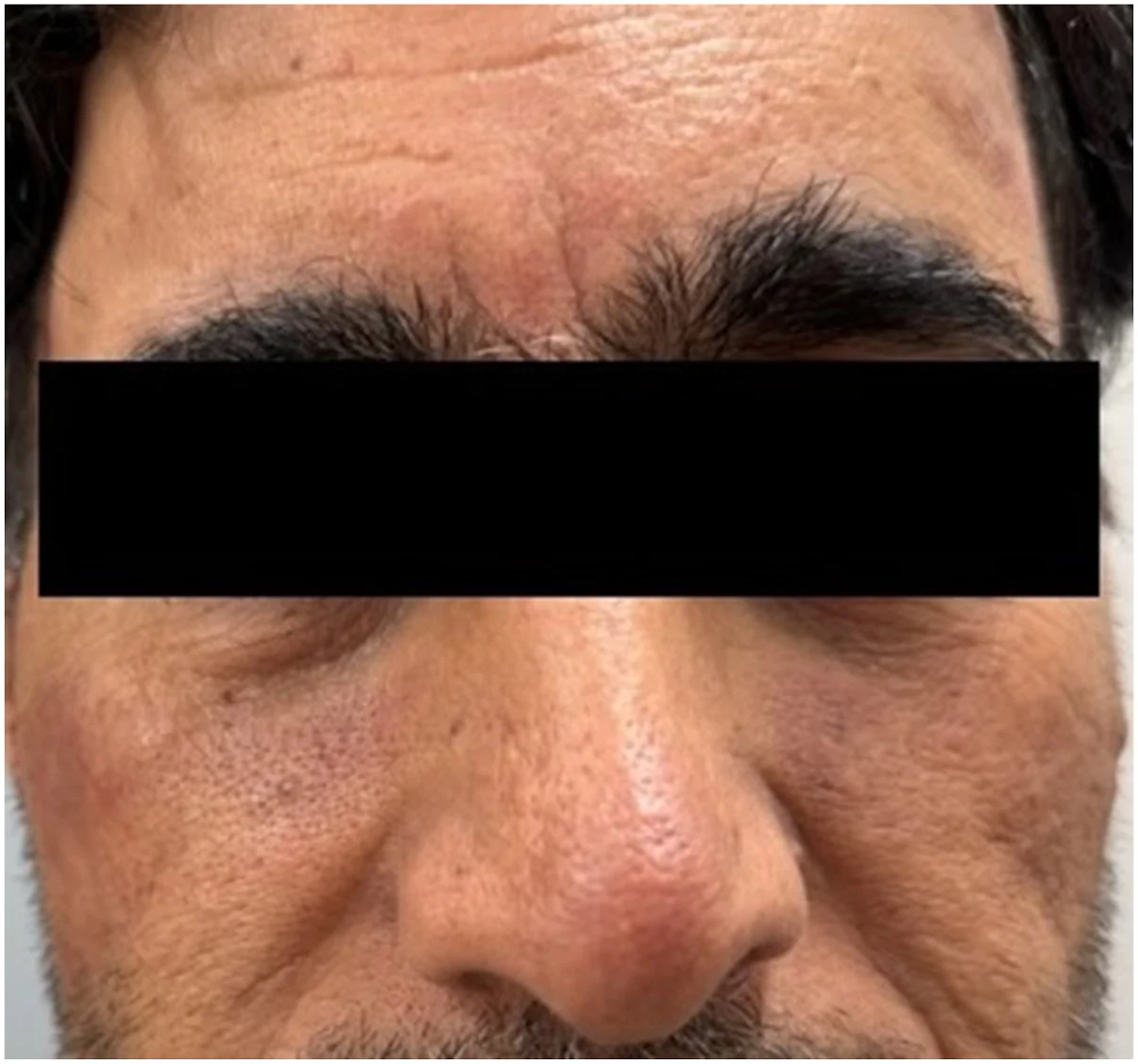
Lymphocytoma cutis. Near-complete resolution of lesions after 12 weeks of upadacitinib 15 mg daily, with residual post-inflammatory hyperpigmentation.

## Discussion

The diagnosis of LC requires a thorough workup to exclude cutaneous lymphoma and other conditions that can produce dense inflammatory infiltrates and should be confirmed by a dermatopathologist experienced in cutaneous lymphoproliferative disorders. On immunohistochemistry, LC reveals polyclonal B-cell populations with both kappa and lambda light chain expression on in situ hybridization.^1^^,^^2^ Our patient's CD20-positive B-cells with polyclonal light-chain expression supported a diagnosis of LC.

Refractory LC is a clinical challenge, particularly when lesions persist despite therapeutic interventions. Our patient had a decade-long history of progressive LC without spontaneous regression and had received several therapies before treatment with upadacitinib resulted in rapid clinical improvement.

Mechanistically, cytokine signaling through the Janus kinase-signal transducer and activator of transcription (JAK-STAT) pathway plays a central role in immune regulation and lymphocyte activation. JAK-mediated signaling is broadly implicated in B-cell proliferation, differentiation, and germinal center responses.^6^^,^^8^ Upadacitinib is a selective JAK1 inhibitor that has been shown to suppress B-cell activation in a dose-dependent manner in experimental models.^6^ Given that LC is a reactive B-cell lymphoproliferative process characterized by prominent germinal centers, inhibition of JAK1-dependent signaling may modulate cytokine-mediated pathways involved in maintenance of reactive lymphoid infiltrates. However, direct evidence is lacking, and its therapeutic effect in this disease remains speculative.

Although JAK inhibitors are increasingly used in inflammatory dermatologic disease, their role in benign cutaneous lymphoid proliferations remains poorly defined. Upadacitinib has been reported to improve refractory facial IgG4 pseudolymphoma, suggesting that JAK1 inhibition may influence reactive lymphoid processes in the skin.^8^ However, its use in LC has not been previously described.

JAK inhibitors carry boxed warnings for malignancy, including lymphoma, and their use in disorders involving lymphoid proliferation warrants careful consideration, as immunomodulatory therapy could obscure or delay recognition of an evolving lymphoid neoplasm. Reports of JAK inhibitor use in patients with cutaneous lymphoid disorders, including cases of misdiagnosed cutaneous T-cell lymphoma, highlight this concern.^2^ Given the potential histopathologic overlap between LC and primary cutaneous B-cell lymphoma, molecular studies for B-cell clonality should be performed before initiating JAK inhibitor therapy. In this case, treatment with upadacitinib was supported by several factors, including the patient's longstanding disease with failure of multiple prior therapies and his preference to avoid additional radiation. Nevertheless, the observed clinical improvement may reflect broader anti-inflammatory or immunomodulatory effects of JAK1 inhibition rather than LC-specific activity.

Although he tolerated treatment well, the limited 12-week follow-up period precludes long-term safety assessment. Ongoing clinicopathologic surveillance, including repeat biopsy if lesions recur or progress, is essential during immunomodulatory therapy as lymphoid infiltrates can evolve over time.^5^

This case identifies selective JAK1 inhibition as a potential therapeutic approach in refractory LC and highlights cytokine-directed signaling pathways as potential targets in persistent disease. Limitations include the single-patient design and inability to determine whether response was specific to JAK1 inhibition versus broader immunosuppressive effects, as well as the absence of mechanistic or biomarker analysis and long-term safety data. Further study is necessary to clarify the safety and efficacy of JAK inhibition in benign cutaneous lymphoid proliferations.

### Declaration of generative AI and AI-assisted technologies in the writing process

Not used.

## Conflicts of interest

None disclosed.
